# Fabry Disease Associated With Myelodysplastic Syndrome: Case Report

**DOI:** 10.1002/ccr3.71055

**Published:** 2025-10-10

**Authors:** Liping Zheng, Zhiwei Wu, Shuzhen Chen, Chunfa Weng, Junwei Huang, Jinzao Chen

**Affiliations:** ^1^ Department of Cardiology The First Hospital of Putian City Putian Fujian China

**Keywords:** case report, Fabry disease, genetic testing, GLA gene, hematological abnormalities, myelodysplastic syndrome

## Abstract

This is the first reported case of Fabry disease (FD) coexisting with myelodysplastic syndrome (MDS). While the coexistence of FD and MDS may be incidental, the case underscores the importance of considering FD in patients with unexplained systemic and hematological abnormalities, particularly those with a family history.

## 1 Introduction

Fabry disease (FD) is a rare X‐linked hereditary lysosomal storage disease resulting from mutations in the α‐galactosidase A (GLA) gene, which causes insufficient enzyme activity, thereby leading to an abnormal accumulation of glycosphingolipids, primarily trihexosylceramide (Gb3) and its derivative Lyso‐Gb3. The gradual accumulation of these substances in multiple organs throughout the body adversely affects normal cell function and tissue health [1, 2, 3, 4]. The clinical manifestations of FD depend on the patient's gender and age and can vary. Typical early symptoms such as angiokeratoma, extremity numbness (pain in the hands and feet), hypohidrosis, gastrointestinal discomfort, and proteinuria are observed in children and adolescents. With disease progression, common complications such as renal failure, heart failure, and cerebrovascular disease afflict adult patients [5, 6].

Myelodysplastic syndrome (MDS) represents a group of clonal hematopoietic disorders caused by inefficient production and abnormal proliferation of blood cells in the bone marrow, causing cytopenias, including anemia, neutropenia, and thrombocytopenia [7, 8]. FD has rarely been associated with hematologic manifestations, and only two cases of FD coexisting with multiple myeloma (MM) have been reported to date. However, there are no documented cases of FD associated with MDS [9, 10]. We hereby report the case of an elderly female patient suffering from pancytopenia, who was later diagnosed with coexisting FD and MDS. Through this case report, the authors aim to raise awareness about the possibility of FD among endocrinologists and hematologists for preventing misdiagnosis or delayed diagnosis.

## 2 Case Description

### 2.1 Patient Presentation

**FIGURE 1:**
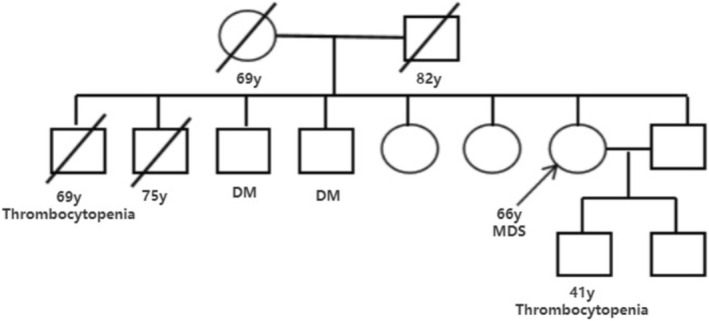
Family pedigree of the patient. The proband (arrow) is a 66‐year‐old female with FD and MDS. Her son shares the same GLA mutation (c.370‐949G>A) and exhibits thrombocytopenia.

A 66‐year‐old Han Chinese woman originally diagnosed with type 2 diabetes for 15 years developed retinal detachment and cataracts, along with peripheral nerve damage in the upper and lower limbs (including sensory and motor impairment), hearing loss, reduced sweating, and intermittent constipation within five years of diagnosis, despite maintaining good glycemic control. After experiencing dizziness and fatigue, she was admitted to the endocrinology department. Blood tests revealed a white blood cell count (WBC) of 2.11 × 10^9^/L (reference range: 3.5–9.5 × 10^9^/L), hemoglobin (HGB) of 33 g/L (reference range: 130–175 g/L), and platelet count (PLT) of 55 × 10^9^/L (reference range: 125–350 × 10^9^/L). She was subsequently referred to the department of hematology to understand the etiology of her pancytopenia. The patient diligently followed the diabetes treatment prescribed and was taking medications including a combination of dapagliflozin, metformin, sitagliptin, and insulin. She had no history of alcohol use or substance abuse. With regard to family history, her brother had died from thrombocytopenia, although the exact cause of his death was unclear. Her son also displayed reduced platelet counts. Other relatives refused to disclose their medical histories (Figure 1).

### 2.2 Physical Examinations

The vital signs of the patient included a height of 155 cm, a weight of 50 kg, a blood pressure of 101/56 mmHg, a pulse rate of 70/min, and a temperature of 36.2°C. The patient exhibited mild pitting edema on the dorsum of the foot, along with peripheral nerve damage in the upper and lower limbs, hearing loss, and reduced sweating.

### 2.3 Laboratory Examinations

Her serum C‐peptide (2 h) level was 0.131 nmol/L (reference range: 0.194–1.326 nmol/L) and the glycosylated hemoglobin level was 6.20% (reference range: 4.2%–6.2%). Renal impairment was indicated through her biochemical test results which were: creatinine (Cr) level of 131 μmol/L (reference range: 44–106 μmol/L), creatine kinase level of 237 U/L (reference range: 38–174 U/L), urine albumin/creatinine ratio of 80.00 mg/g (reference range: 0–30 mg/g), microalbumin 35.6 mg/L (reference range: 0–19 mg/L), and α1‐microglobulin 12.20 mg/L (reference range: 0–6 mg/L). Additionally, her renal autoantibody test yielded negative results. Autoimmune disease‐related tests, direct antiglobulin test, and serum acidification hemolysis test all tested negative; however, her complement C3 level was 0.65 g/L (reference range: 0.9–1.8 g/L). Liver function, coagulation function, and thyroid function were normal. Heart failure accompanied by myocardial injury was indicated from her N‐terminal pro‐B‐type natriuretic peptide (NT‐ProBNP) level of 2428.64 pg/mL (reference range: 0–900 pg/mL), and the high‐sensitivity troponin I level of 0.179 μg/L (reference range: 0–0.03 μg/L).

### 2.4 Diagnosis Process

**FIGURE 2:**
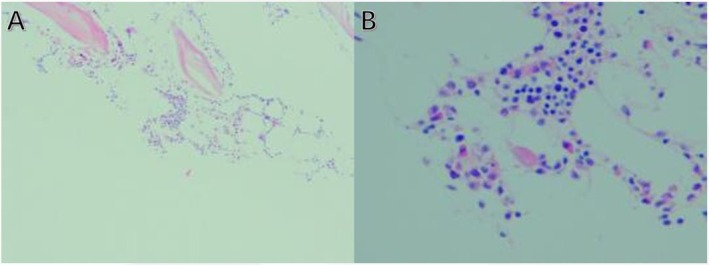
Bone marrow biopsy findings. The biopsy reveals reduced cellularity with vacuolization and megaloblastic features, consistent with MDS.

**FIGURE 3:**
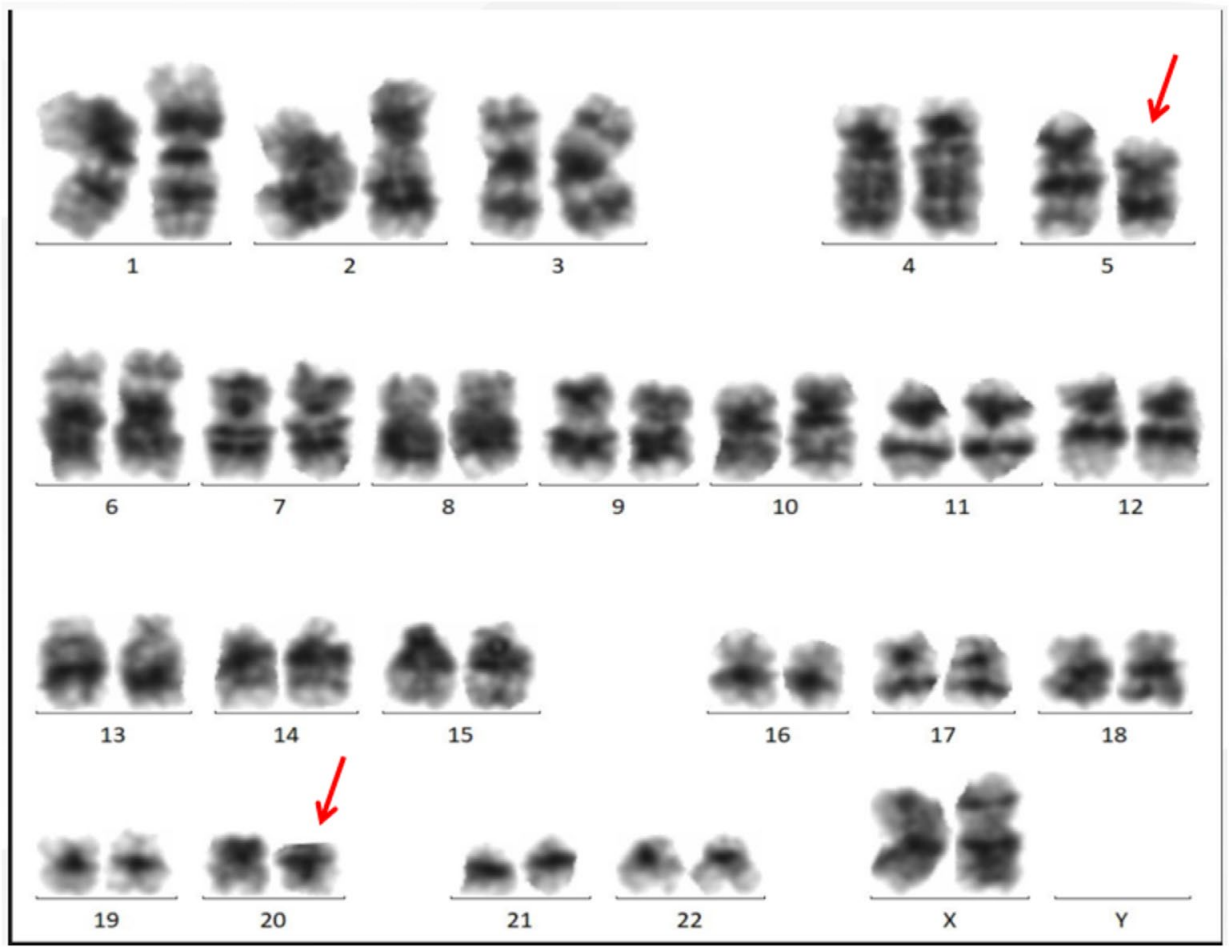
Cytogenetic analysis of the bone marrow. Karyotyping shows del(5q) and del(20q), confirming the diagnosis of MDS.

The hematologist conducted a series of diagnostic tests for her pancytopenia. Her peripheral blood smear revealed the presence of 1.0% promyelocytes and 1.0% myelocytes. These immature cell types, which are typically found in the bone marrow, only appear in peripheral blood under certain pathological conditions, making this finding significant. The percentage of reticulocytes was elevated, but the reticulocyte count was normal. Specifically, elevated proportions of immature reticulocytes (27.4%, reference range: 0%–25%) and those with high fluorescence intensity (11.3%, reference range: 0%–5%) were observed. These findings suggested increased erythropoietic activity, possibly indicating an active bone marrow response to anemia. A subsequent bone marrow aspiration revealed a significant reduction in the proliferation of nucleated cells. Further observation of the bone marrow cells showed signs of abnormal cellularity with reduced cellular density and vacuolization. Although initial bone marrow findings, including reduced cellularity and vacuolization, suggested the possibility of megaloblastic anemia, classic morphological features such as nuclear‐cytoplasmic asynchrony and hypersegmented neutrophils were absent. Furthermore, elevated serum folate and vitamin B12 levels did not support this diagnosis. As shown in Figure 2, the iliac bone marrow biopsy (H&E staining) showed reduced cellularity (10%–20%) and a reduced ratio of granulocytes to erythrocytes, with a slight increase in the proportion of immature cells across all three cell lineages. The megakaryocytes primarily exhibited lobulated nuclei. Evidence of reticular fiber staining (MF‐0 grade) was also noted. These findings, though indicative of bone marrow dysplasia, were more suggestive of myelodysplastic syndrome (MDS) rather than megaloblastic anemia. Finally, a detailed karyotyping of the bone marrow cells of the patient demonstrated multiple significant mutations in the chromosomes, with a karyotype of 46, XX, del (5) (q31q35), del (20) (q11.2q13.3), indicating the deletion of chromosomes 5 and 20 (Figure 3). The patient was finally diagnosed with MDS (5q‐syndrome).

**FIGURE 4:**
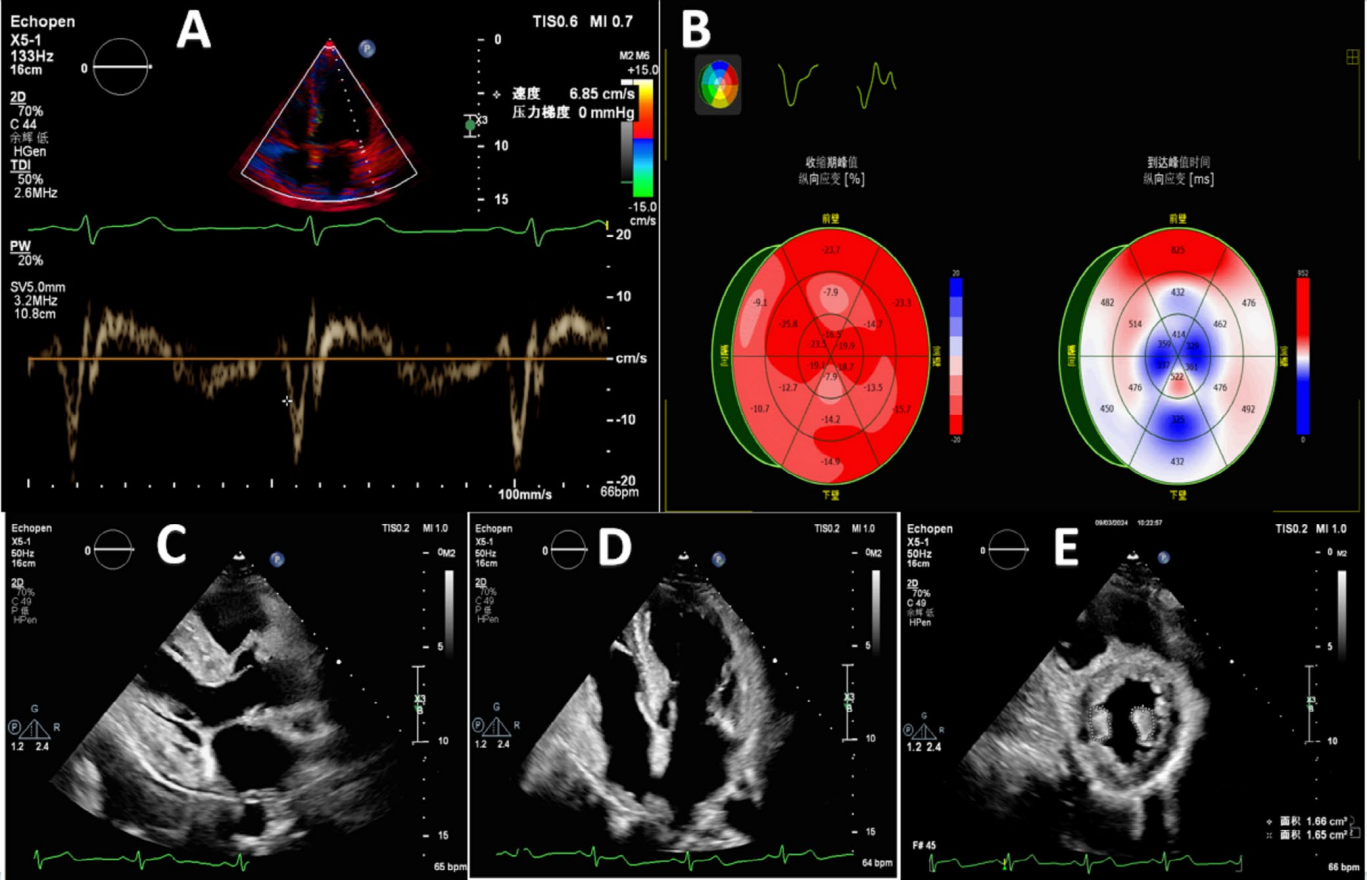
Representative images of noninvasive cardiac modalities in patient. (A) Mitral inflow Doppler echocardiography: Shows reduced diastolic velocities, suggestive of diastolic dysfunction; (B) Longitudinal strain observed on speckle tracking echocardiography; (C, D) Parasternal long axis and apical long axis show symmetrical hypertrophy. The parasternal long axis and apical long axis demonstrated symmetrical hypertrophy; (E) Hypertrophy of papillary muscles in parasternal short axis.

**FIGURE 5:**
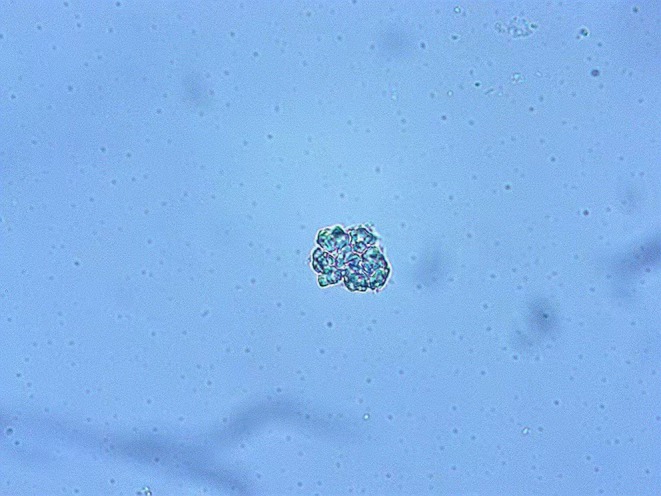
Urinary sediment test findings. The presence of mulberry bodies is indicative of FD.

**FIGURE 6:**
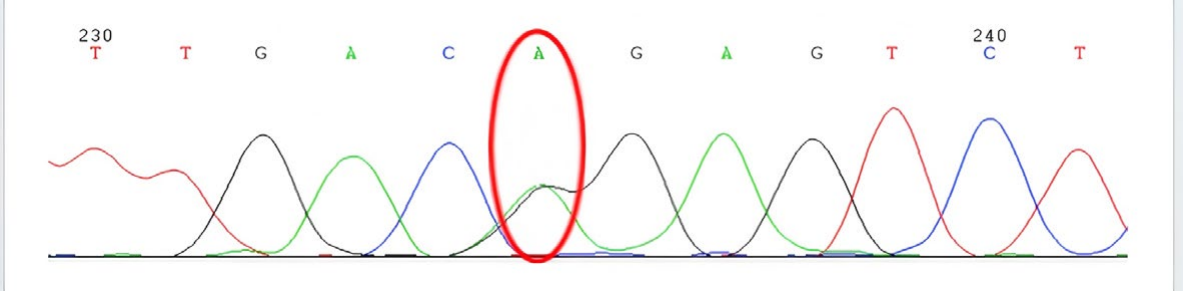
Genetic analysis of the GLA gene. A novel intronic variant, c.370‐949G>A, in the GLA gene was identified and classified as a variant of uncertain significance (VUS).

Notably, the findings of echocardiography indicated: an interventricular septum (IVS) thickness of 15 mm (reference value: ≤ 11 mm); a left ventricular posterior wall (LVPW) thickness of 13 mm (reference value: ≤ 11 mm); thickening of the aortic valve leaflets; calcification of the posterior mitral annulus; patent foramen ovale; and impaired systolic and diastolic function (Figure 4). In addition, the urinary sediment test revealed the presence of mulberry bodies (Figure 5). There was a reduction in her leukocyte GLA enzyme activity level (0.7 μmol/L/h; normal range: 2.2–17.65 μmol/L/h), while the plasma Lyso‐globotriaosylsphingosine (Lyso‐Gb3), a biomarker for FD, was within the normal range (< 0.55 ng/mL; reference value: < 1.11 ng/mL). A novel intronic variant in the GLA gene (NM_000169), c.370‐949G>A (GRCh37: chrX:100657746), was identified by Sanger sequencing. This variant is not reported in ClinVar, gnomAD, or dbSNP and is therefore classified as a variant of uncertain significance (VUS). At present, there is no evidence that it contributes to the patient's hematological abnormalities, and it is reported here as an incidental finding (Figure 6). These findings support a diagnosis of heterozygous Fabry disease. It is important to note that, while several family members have a history of low platelet counts, the presence of this mutation does not necessarily explain the thrombocytopenia. The patient's son also exhibited the same mutation along with thrombocytopenia; however, the relationship between this mutation and thrombocytopenia remains speculative. Thrombocytopenia is not a common feature of FD, and other genetic factors or conditions may contribute to this hematologic abnormality. Further investigation, including screening for common thrombocytopenia‐related mutations (such as GATA1, RUNX1, ETV6, ANKRD26), would be important to fully understand the etiology of thrombocytopenia in this family. A clinical diagnosis of FD was made based on the family history, the detection of mulberry bodies in the urinary sediment test, and the characteristic findings on cardiac examination. Thus, FD can be considered to play a significant role in the cardiac manifestations observed in this patient, although its role in the hematological abnormalities remains uncertain.

## 3 Discussion

The incidence of Fabry disease (FD) is considered to be low, affecting approximately 1 in 40,000–117,000 individuals, with a greater proportion of the affected population being male. However, recent newborn screening studies suggest a potentially higher incidence of FD, especially in atypical forms. Female carriers tend to exhibit milder symptoms, though their prognosis is still impacted [11, 12, 13, 14]. In the early stages of this progressive genetic metabolic disorder, cellular dysfunction and microvascular disease result from the accumulation of glycosphingolipids (such as ceramide dihexoside) in lysosomes [15]. This disease is caused by a deficiency or insufficient activity of the lysosomal enzyme α‐galactosidase A (α‐GalA), leading to ineffective degradation of glycosphingolipids. Various cell types, including endothelial cells in capillaries, podocytes in the kidneys, tubular and glomerular endothelial cells, cardiomyocytes, fibroblasts, and neurons in both the central and peripheral nervous systems, are affected by the accumulation of these metabolic products [16]. The pathological changes associated with FD may manifest as early as infancy or even in utero. However, clinical symptoms often remain subtle in the early years, with the disease progressing insidiously. As patients age, they develop symptoms such as burning pain in the limbs, fever, reduced exercise tolerance, and fatigue, typically between the ages of 3 and 10 years. Female patients usually experience symptoms later than male patients, and their disease tends to be less severe due to the X‐linked inheritance pattern of FD [17]. As the disease progresses, multiple organ systems are affected. Glycosphingolipid storage leads to cellular dysfunction and triggers a series of pathological events, including cell death, metabolic dysregulation, microvascular damage, dysfunction of K (Ca) 3.1 channels in endothelial cells, and increased oxidative stress [18]. Additionally, impaired maturation of autophagosomes exacerbates the condition, reducing the ability of cells to clear damaged proteins and metabolic waste [19]. These processes eventually lead to irreversible fibrosis, particularly in the heart and kidneys, contributing to organ failure in FD. Over time, patients often suffer from severe cardiovascular and cerebrovascular complications, progressing to end‐stage renal disease, which significantly reduces life expectancy. Untreated patients commonly die from heart failure, arrhythmia, or stroke in early adulthood.

Currently, very few reports document hematological disorders caused by FD. Only two cases of FD coexisting with multiple myeloma (MM) have been reported, and no documented cases exist of FD coexisting with thrombocytopenia, especially in the context of myelodysplastic syndrome (MDS) [9, 10]. MDS is typically associated with mutations in hematopoietic stem cells, disrupting normal blood cell maturation and leading to the accumulation of abnormal, immature cells in the bone marrow [20, 21, 22, 23].

In the current case, a 66‐year‐old female patient was diagnosed with FD, and a 5q deletion was identified on bone marrow examination, which is a common chromosomal abnormality associated with myelodysplastic syndrome (MDS), accompanied by thrombocytopenia. Several family members, including her son, also exhibited thrombocytopenia. However, it is important to note that there is no direct evidence linking FD with hematological abnormalities in the literature, and the coexistence of these two conditions may be incidental. Potential mechanisms may exist, but further investigation is warranted. Specifically, the c.370‐949G>A mutation identified in this case is a novel intronic variant that has not been previously reported in the literature. Due to the lack of functional data (such as splicing assays or RNA analyses) supporting its pathogenicity, this variant should currently be classified as a variant of uncertain significance (VUS). Its potential role in the patient's phenotype remains speculative and warrants further investigation [24]. Hematopoietic stem cells are highly dependent on lysosomal function, and the accumulation of glycosphingolipids in lysosomes could impair stem cell function [25]. Moreover, macrophages in the bone marrow play a critical role in regulating blood cell production, and the accumulation of glycosphingolipids could inhibit macrophage function, disrupting the bone marrow microenvironment [26]. Chronic low‐grade inflammation, frequently observed in FD, may also contribute to hematopoietic abnormalities by inhibiting the proliferation of hematopoietic stem cells [27]. However, the role of FD in hematological manifestations remains unclear and requires further exploration. In summary, FD is a rare disease with subtle symptoms in its early stages, often misdiagnosed as common diseases like diabetic neuropathy, cardiomyopathy, or renal insufficiency. This case highlights the importance of considering rare diseases when faced with unexplained multi‐system symptoms, particularly in patients with a family history. Genetic testing plays a key role in preventing misdiagnosis.

In recent years, the outlook for FD treatment has improved with enzyme replacement therapy (ERT), which can delay or partially reverse organ damage, especially in the kidneys and heart. However, optimal outcomes depend on early intervention. Unfortunately, this patient declined further targeted therapy due to financial limitations.

## Author Contributions

**Liping Zheng:** conceptualization, validation, visualization. **Zhiwei Wu:** project administration, resources, software, writing – original draft. **Shuzhen Chen:** data curation. **Chunfa Weng:** funding acquisition, software. **Junwei Huang:** formal analysis. **Jinzao Chen:** writing – review and editing.

## Consent

Written informed consent was obtained from the patient for the publication of this case report and accompanying images.

## Conflicts of Interest

The authors declare no conflicts of interest.

## Data Availability

All data supporting the findings of this case report are included in this article. Further details are available from the corresponding author upon reasonable request.

## References

[1] D. P. Germain , “Fabry Disease,” Orphanet Journal of Rare Diseases 5 (2010): 30. 21092187 10.1186/1750-1172-5-30 PMC3009617

[2] A. Ortiz , D. P. Germain , R. J. Desnick , et al., “Fabry Disease Revisited: Management and Treatment Recommendations for Adult Patients,” Molecular Genetics and Metabolism 123, no. 4 (2018): 416–427. 29530533 10.1016/j.ymgme.2018.02.014

[3] R. O. Brady , A. E. Gal , R. M. Bradley , E. Martensson , A. L. Warshaw , and L. Laster , “Enzymatic Defect in Fabry's Disease. Ceramidetrihexosidase Deficiency,” New England Journal of Medicine 276, no. 21 (1967): 1163–1167. 6023233 10.1056/NEJM196705252762101

[4] C. M. Eng , J. Fletcher , W. R. Wilcox , et al., “Fabry Disease: Baseline Medical Characteristics of a Cohort of 1765 Males and Females in the Fabry Registry,” Journal of Inherited Metabolic Disease 30, no. 2 (2007): 184–192. 17347915 10.1007/s10545-007-0521-2

[5] R. J. Hopkin , J. Bissler , M. Banikazemi , et al., “Characterization of Fabry Disease in 352 Pediatric Patients in the Fabry Registry,” Pediatric Research 64, no. 5 (2008): 550–555. 18596579 10.1203/PDR.0b013e318183f132

[6] W. R. Wilcox , J. P. Oliveira , R. J. Hopkin , et al., “Females With Fabry Disease Frequently Have Major Organ Involvement: Lessons From the Fabry Registry,” Molecular Genetics and Metabolism 93, no. 2 (2008): 112–128. 18037317 10.1016/j.ymgme.2007.09.013

[7] D. A. Galton , “The Myelodysplastic Syndrome,” BMJ 299, no. 6699 (1989): 582. 2508812 10.1136/bmj.299.6699.582 PMC1837454

[8] C. V. Dang , “Myelodysplastic Syndrome,” JAMA 267, no. 15 (1992): 2077–2080. 1552643 10.1001/jama.267.15.2077

[9] K. Adachi , H. Tokuyama , Y. Oshima , et al., “Fabry Disease Associated With Multiple Myeloma: A Case Report,” CEN Case Reports 11, no. 1 (2022): 146–153. 34529243 10.1007/s13730-021-00613-x PMC8810996

[10] K. Taguchi , A. Moriyama , G. Kodama , Y. Nakayama , and K. Fukami , “The Coexistence of Multiple Myeloma‐Associated Amyloid Light‐Chain Amyloidosis and Fabry Disease in a Hemodialysis Patient,” Internal Medicine 56, no. 7 (2017): 841–846. 28381753 10.2169/internalmedicine.56.7623 PMC5457930

[11] W. L. Hwu , Y. H. Chien , N. C. Lee , et al., “Newborn Screening for Fabry Disease in Taiwan Reveals a High Incidence of the Later‐Onset GLA Mutation c.936+919G>A (IVS4+919G>A),” Human Mutation 30, no. 10 (2009): 1397–1405. 19621417 10.1002/humu.21074 PMC2769558

[12] T. Inoue , K. Hattori , K. Ihara , A. Ishii , K. Nakamura , and S. Hirose , “Newborn Screening for Fabry Disease in Japan: Prevalence and Genotypes of Fabry Disease in a Pilot Study,” Journal of Human Genetics 58, no. 8 (2013): 548–552. 23677059 10.1038/jhg.2013.48

[13] P. J. Meikle , J. J. Hopwood , A. E. Clague , and W. F. Carey , “Prevalence of Lysosomal Storage Disorders,” JAMA 281, no. 3 (1999): 249–254. 9918480 10.1001/jama.281.3.249

[14] M. Spada , S. Pagliardini , M. Yasuda , et al., “High Incidence of Later‐Onset Fabry Disease Revealed by Newborn Screening,” American Journal of Human Genetics 79, no. 1 (2006): 31–40. 16773563 10.1086/504601 PMC1474133

[15] B. L. Thurberg , J. T. Fallon , R. Mitchell , T. Aretz , R. E. Gordon , and M. W. O'Callaghan , “Cardiac Microvascular Pathology in Fabry Disease: Evaluation of Endomyocardial Biopsies Before and After Enzyme Replacement Therapy,” Circulation 119, no. 19 (2009): 2561–2567. 19414635 10.1161/CIRCULATIONAHA.108.841494

[16] R. O. Brady and R. Schiffmann , “Enzyme‐Replacement Therapy for Metabolic Storage Disorders,” Lancet Neurology 3, no. 12 (2004): 752–756. 15556808 10.1016/S1474-4422(04)00938-X

[17] Y. A. Zarate and R. J. Hopkin , “Fabry's Disease,” Lancet 372, no. 9647 (2008): 1427–1435. 18940466 10.1016/S0140-6736(08)61589-5

[18] B. Breiden and K. Sandhoff , “Lysosomal Glycosphingolipid Storage Diseases,” Annual Review of Biochemistry 88 (2019): 461–485. 10.1146/annurev-biochem-013118-111518 31220974

[19] M. Chévrier , N. Brakch , L. Céline , et al., “Autophagosome Maturation Is Impaired in Fabry Disease,” Autophagy 6, no. 5 (2010): 589–599. 20431343 10.4161/auto.6.5.11943

[20] T. N. Tanaka and R. Bejar , “MDS Overlap Disorders and Diagnostic Boundaries,” Blood 133, no. 10 (2019): 1086–1095. 30670443 10.1182/blood-2018-10-844670

[21] E. Estey , R. P. Hasserjian , and H. Döhner , “Distinguishing AML From MDS: A Fixed Blast Percentage May No Longer Be Optimal,” Blood 139, no. 3 (2022): 323–332. 34111285 10.1182/blood.2021011304 PMC8832464

[22] C. Aul , D. T. Bowen , and Y. Yoshida , “Pathogenesis, Etiology and Epidemiology of Myelodysplastic Syndromes,” Haematologica 83, no. 1 (1998): 71–86. 9542325

[23] C. Wang , Y. Yang , S. Gao , et al., “Immune Dysregulation in Myelodysplastic Syndrome: Clinical Features, Pathogenesis and Therapeutic Strategies,” Critical Reviews in Oncology/Hematology 122 (2018): 123–132. 29458780 10.1016/j.critrevonc.2017.12.013

[24] P. Li , Y. Xi , Y. Zhang , et al., “GLA Mutations Suppress Autophagy and Stimulate Lysosome Generation in Fabry Disease,” Cells 13, no. 5 (2024): 437. 38474401 10.3390/cells13050437 PMC10930447

[25] D. Loeffler , A. Wehling , F. Schneiter , et al., “Asymmetric Lysosome Inheritance Predicts Activation of Haematopoietic Stem Cells,” Nature 573, no. 7774 (2019): 426–429. 31485073 10.1038/s41586-019-1531-6

[26] A. Ersek , K. Xu , A. Antonopoulos , et al., “Glycosphingolipid Synthesis Inhibition Limits Osteoclast Activation and Myeloma Bone Disease,” Journal of Clinical Investigation 125, no. 6 (2015): 2279–2292. 25915583 10.1172/JCI59987 PMC4518690

[27] B. Laffer , M. Lenders , E. Ehlers‐Jeske , K. Heidenreich , E. Brand , and J. Köhl , “Complement Activation and Cellular Inflammation in Fabry Disease Patients Despite Enzyme Replacement Therapy,” Frontiers in Immunology 15 (2024): 1307558. 38304433 10.3389/fimmu.2024.1307558 PMC10830671

